# Being vulnerable

**DOI:** 10.7554/eLife.59285

**Published:** 2020-06-15

**Authors:** Jasper Elan Hunt

**Affiliations:** Department of Physiology, Anatomy and Genetics, University of OxfordOxfordUnited Kingdom

**Keywords:** COVID-19 and the Research Community, early-career researchers, mental health, careers in science, grad school

## Abstract

Early-career researchers feel discouraged from exposing vulnerability even during a global crisis.

When governments began restricting travel in response to the COVID-19 pandemic, I was faced with a decision: do I travel home to be with my family, thousands of miles away in Wyoming, or do I stay in my college-owned accommodation in the UK, close to the lab?

The first considerations on my mind were pragmatic: my university might reopen research facilities long before travel restrictions are eased, being abroad might prevent me from returning to lab work in a timely manner, it would be expensive to book an international flight on short notice, and what could happen to my visa if I were to leave the country now? There only seemed to be one option in this situation; I would not be returning home.

Like many other early-career researchers (Kong, 2020; Mahul-Mellier, 2020), I grossly underestimated the difficulties of remote working. I tried to continue working at normal productivity levels even as my mental health and motivation deteriorated. I took on new projects and commitments to maintain output while my work-life balance vanished. But as I adjusted to the realities of an unprecedented situation, I was spending more hours working while I was accomplishing less. Even as I floundered, I felt there was no one I could confide in.

Under ordinary circumstances, many early-career researchers grapple with feelings of self-doubt and inadequacy. Despite evidence of one’s objective successes, it is easy to feel as though one is always trying to catch up from behind, out of one's depth, and soon to be “found out” as an impostor. This phenomenon, impostor syndrome, has traditionally been associated with early-career academics, and might disproportionately affect women (Vaughn et al., 2020) and other underrepresented demographics in academia. Even under the best of circumstances, the fear of being “discovered” as an impostor is enough, in itself, to discourage many from sharing their anxieties with others. But as the research community reels from a global crisis, finding social support is as important as ever.

It is easy for the latent feelings of inadequacy associated with impostor syndrome to compound with the challenges of life during the COVID-19 pandemic. When I was not as productive as I expected, these feelings quickly snowballed into self-doubt: why wasn’t I working harder? If I can’t find the motivation to work on fairly straightforward analyses at home, what hope do I have of earning my PhD, let alone becoming a tenure-track professor? It is easy to overlook or brush off the successes of the past when confronted with immediate challenges and I am certain that many of my colleagues – especially graduate students, postdocs and new principal investigators – have felt similarly helpless at times during recent months. The lockdown has been stressful for many of us in varied, individualistic ways. The difficulties of an unprecedented situation have changed our routines in profound ways and introduced difficulties few of us could have anticipated. For those contending with shifts in domestic and caring responsibilities and abrupt changes in income, the stress and uncertainty has been even greater. It is no wonder that many early-career researchers have encountered challenges to their mental health. But in these moments of self-doubt, who can an early-career researcher turn to?

There is an enormous pressure for early-career researchers to succeed. The number of PhDs awarded has vastly outstripped the number of positions available for them in the academic workforce and the number of postdocs vastly outnumbers the principal investigator positions available (Bourne, 2013). Those who move into other careers after a PhD or postdoc will have gained invaluable skills along the way (Marder, 2014), but it remains true that many research trainees wish to continue with careers in science. Those who “win” coveted slots in PhD programmes or tenure-track faculty positions have beaten the odds of a difficult market, and the pressure to succeed is immense.

Is success compatible with vulnerability? At times, it can feel as though admitting weakness is not an option. Between an uncertain academic job market, fierce competition for grant applications, and the myriad other professional challenges faced by early-career scientists, it can feel difficult to justify slowing the pace of one’s work. Many might wonder, will hiring committees or grant panels look favourably upon a CV with a sparse publication record for this year? These sorts of concerns can be amplified for early-career researchers from underrepresented minorities, who may already feel they are fighting against the current of institutional barriers.

The present crisis has highlighted a common perception: that vulnerability can be lethal in a hyper-competitive research market. Yet pausing to care for one’s mental health is important for everyone, including those wanting to perform good science. In the same way that the race to publish novel findings disincentivises careful science (Marder, 2015), the scientific community is doing itself a disservice by pressuring its fledgling scientists to prioritise research output over personal well-being.

Science is a creative endeavour. Careful, elegant experiments rarely arise from the mentality of putting one’s nose to the grindstone and persevering. To produce insightful, reproducible experiments that will withstand the tests of time, we should encourage our early-career researchers to prioritise their mental health and emotional well-being. If we value our scientists, our science will surely benefit.
